# Antidiabetic, antioxidant, and anti-obesity effects of phenylthio-ethyl benzoate derivatives, and molecular docking study regarding α-amylase enzyme

**DOI:** 10.1038/s41598-022-07188-2

**Published:** 2022-02-24

**Authors:** Nidal Jaradat, Ahmad Khasati, Maram Hawi, Mohammed Hawash, Suhaib Shekfeh, Mohammad Qneibi, Ahmad M. Eid, Mohammad Arar, Mohammed T. Qaoud

**Affiliations:** 1 Department of Pharmacy, Faculty of Medicine and Health Sciences, An-Najah National University, P.O. Box 7, 00970 Nablus, Palestine; 2 Chemometrics and Analytical Chemistry, Modern Testing Services, Povinostr. 52, 86153 Augsburg, Germany; 3 Department of Biomedical Sciences, Faculty of Medicine and Health Sciences, An-Najah National University, 00970 Nablus, Palestine; 4 Department of Pharmaceutical Chemistry, Faculty of Pharmacy, Gazi University, 06330 Etiler, Ankara, Turkey

**Keywords:** Biological techniques, Chemical biology, Drug discovery, Diseases

## Abstract

In addition to their wide therapeutic application, benzoates and benzoic acid derivatives are the most commonly utilized food preservatives. The purpose of this study was to estimate the antioxidant, anti-diabetic, and anti-obesity activities of four 2-(phenylthio)-ethyl benzoate derivatives utilizing standard biomedical assays. The results revealed that the **2a** compound has potent antidiabetic activity through the inhibition of α-amylase and α-glycosidase with IC_50_ doses of 3.57 ± 1.08 and 10.09 ± 0.70 µg/ml, respectively, compared with the positive control acarbose (IC_50_ = 6.47 and 44.79 µg/ml), respectively. In addition, by utilizing the β-carotene linoleic acid and DPPH methods, the **2a** compound showed the highest antioxidant activity compared with positive controls. Moreover, the **2a** compound showed potential anti-lipase activity with an IC_50_ dose of 107.95 ± 1.88 µg/ml compared to orlistat (IC_50_ = 25.01 ± 0.78 µg/ml). A molecular docking study was used to understand the interactions between four derivatives of (2-(phenylthio)-ethyl benzoate with α-amylase binding pocket. The present study concludes that the **2a** compound could be exploited for further antidiabetic, antioxidant, and anti-obesity preclinical and clinical tests and design suitable pharmaceutical forms to treat these global health problems.

## Introduction

Diabetes mellitus is a chronic disease that presents as both fasting and postprandial hyperglycemia, with disruptions in the metabolizing of carbohydrates and proteins. Chronic hyperglycemia can cause impairment to all organs of the human body by decreasing blood vessels' elasticity and narrowing them, impeding blood flow. This can cause a reduction in blood and oxygen supplies to all organs, increase the risk of hypertension and cause multiple damages to large and small blood vessels. Thus, it can be implicated in many macrovascular and microvascular illnesses, including heart attack, stroke, peripheral arterial disease, retinopathy, neuropathy, and nephropathy^1,2^. In recent years, there has been a rise in the global incidence of diabetes. According to the International Diabetes Federation, the estimated number of individuals with diabetes reached 30 million in 1985 and is expected to reach 380 million by 2025^3^.

Antioxidant agents are free radical scavenging molecules that may prevent or delay several forms of cell damage by neutralizing the harmful free radicals in the living cells and thereby preventing or delaying various diseases^4–7^.

According to the World Health Organization, obesity is one of the most prevalent health concerns nowadays, and it is believed to be one of the major causes of cancer and a variety of other ailments^8^. In general, obesity is often caused by a number of variables, beginning with a high caloric food intake and inadequate physical activity, and it may be influenced by genetic susceptibility^9,10^.

**Figure 1:**
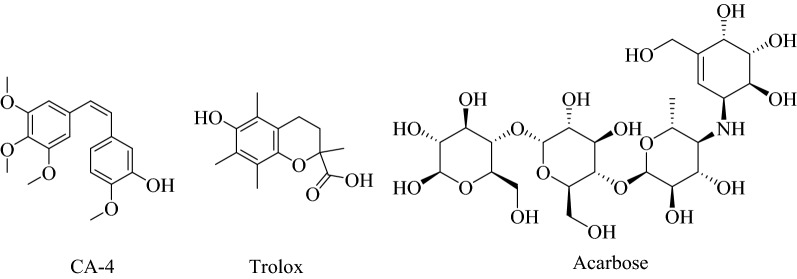
Structures of bioactive compounds containing a hydroxyl group.

Many structures containing hydroxyl functional groups are considered pharmacologically active toward different biological targets, such as combretastatin A-4 (CA-4), which has potent anticancer activity^11^, trolox has a powerful antioxidant effect, and acarbose has antidiabetic property (Fig. 1).

Regarding these structures, the current investigation aims to evaluate new molecules containing a hydroxyl functional group prepared from the esterification of thio-acid with benzoic acid derivatives, including benzoic acid (**2a**), 2-hydroxybenzoic acid (**2b**), 3-hydroxybenzoic acid (**2c**), and 4-hydroxybenzoic acid (**2d**) with 2-thiophenylethanol as antidiabetic, anti-obesity, and antioxidant agents by the inhibition of core metabolic enzymes implicated in hyperglycemia (α-amylase and α-glucosidase), lipids metabolism and oxidative stress.

## Materials and methods

### Chemicals and instruments

**Figure 2:**
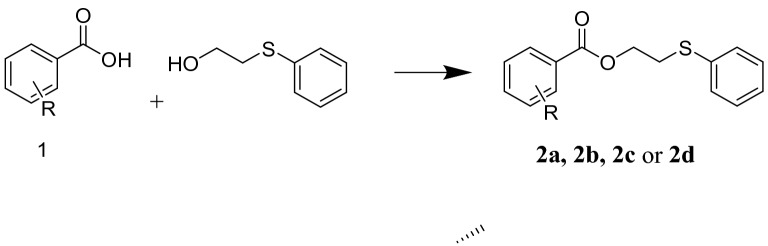
The reaction of benzoic acid derivatives with thiophenyl ethanol. (When R = H presents compound **2a**, R = 2-OH presents **2b**, R = 3-OH presents **2c,** and R = 4-OH presents **2d**).

The following chemicals and biological materials were purchased from Frutarom (UK): β-carotene, DPPH, Na_2_HPO_4_/NaH_2_PO_4_, NaCl, gallic acid, trolox, methanol, starch, porcine pancreatic amylase, lipase, glucosidase, dinitrosalicylic acid (DNSA), Na_2_CO_3_, dimethyl sulfoxide (DMSO), and acarbose. The evaluated compounds **2a**–**2d** were synthesized previously by our team, as outlined in Fig. 2^12^.

### α-Amylase inhibitory activity

The α-amylase inhibitory evaluation was established following the Wickramaratne et al. procedure^13^ with minor changes. Working solutions with concentrations of 10, 50, 100, 500, and 1000 µg/ml were produced from our synthesized compounds, diluting them with a buffer of Na_2_HPO_4_/NaH_2_PO_4_ (0.02 M), NaCl (0.006 M) at pH 6.9 and then brined up to 10 ml using a 10 ml volumetric flask. The acarbose was considered a positive control and was established following the same previous steps utilized for the synthesized compounds. As percent inhibition, the enzyme inhibitory potential was expressed, and the following equation was utilized to estimate the IC_50_ dose for the samples^14^.

$$\%a-Amylase\ Inhibiton=\frac{Abs100\%\ control-Abs\ Sample}{Abs\ 100\%\ Control}\times 100$$

### α-Glucosidase activity

The inhibition of α-glucosidase activity by the synthesized molecules was measured. The α-glucosidase inhibitory activity is expressed as percentages of inhibition, which was calculated using the following formula:

$$ {\text{Inhibitory}}\,{\text{effect }}\% \, = \, \left( {A_{b} - \, A_{s} } \right)/ \, A_{b} *100 \, \% $$

where A_b_ and A_s_ are the absorbance values of the blank (containing PBS, α-glucosidase, and PNPG, the colored substrate of glucosidase) and the tested samples containing PBS, samples, α-glycosidase, and PNPG, respectively.

The α-glucosidase activity was measured using 10 and 20 mg/ml of the synthesized molecules. Each concentration was recorded by using 1, 3, 6, 9, and 12 mm PNPG. The inhibitory pattern was assessed using a Lineweaver–Burk plot. The constant Ki of enzyme inhibitory effect was determined^15^.

### Antioxidant activity

#### DPPH method

Four synthetic compounds were tested for the efficiency of scavenging free radicals matched with trolox and gallic acid as a basic. From the compounds, 1 mg/ml concentration solutions in methanol were prepared, and the solutions were used to prepare concentrations of 5, 10, 20, 30, 40, and 50 μg/ml. The DPPH reagent (0.002% w/v) was dissolved in methanol before mixed with working concentrations in ratios of 1:1:1 (compound:DPPH:methanol). The methanol solution was adopted as a blank. All the solutions were incubated for 30 min at room temperature in the dark. When the antioxidant compound reacts with DPPH, which can donate hydrogen, it is reduced the color changes from deep violet to light. Absorbance values were estimated by using a UV–Vis spectrophotometer at a wavelength of 517 nm. The DPPH inhibition percentage of all tested compound, trolox and gallic acid, was estimated according to the formula:

$$ {\text{Inhibition}}\,{\text{ percentage }} = \, \left( {A_{b} - A_{s} } \right)/A_{b} \times 100\% , $$

where A_b_ is blank absorbance, and A_S_ is sample absorbance. The IC_50_ values for tested synthetic compounds, trolox and gallic acid were determined from the curves^16^.

#### β-Carotene method

The activity of the synthesized compounds was assessed via performing a modified assay of Gazzani and Miller^17^. The method was based on the coupled oxidation of β-carotene and linoleic acid emulsion. The bleaching mechanism of β-carotene is produced from the hydro-peroxides created from linoleic acid^18^. During the oxidation process, the characteristic orange color of β-carotene and the chromophore will be lost. The presence of antioxidants can hinder the extent of the β-carotene bleaching. Briefly, 1 mg of β-carotene was dissolved in 2 ml of chloroform, 20 mg of linoleic acid, and 200 mg of Tween 20. A rotary evaporator entirely vaporized chloroform at a temperature of less than 30 °C under reduced pressure. Then 200 ml of distilled water saturated with oxygen was added to the flask, which was shaken vigorously for 30 min. A sample (5 ml) of the prepared emulsion was transferred to a series of tubes containing 0.1 ml of the synthesized compounds or tocopherol (2 mg/ml).

The control sample was prepared in the same way but without added antioxidants. Each sample type was prepared in triplicate. The samples were inserted in a water bath at 50 °C for 2 h. The absorbance of the samples was measured using a spectrophotometer at a wavelength of 470 nm, immediately after the preparation of the samples, and at 15-min intervals until the end (t = 120 min) of the experiment.

### Anti-lipase activity

The lipase inhibitory activity was conducted by following the method in the references with minor modifications^19–21^. The prepared synthesized compounds solutions (1 mg/ml) were diluted with 10% DMSO to produce five different dilutions (0.2, 0.4, 0.6, 0.8, and 1 mg/ml). Orlistat anti-obesity drug was considered a positive control in this protocol and was then tested following the same steps used previously. A Tris–HCl buffer was used to zero UV–Vis spectrophotometer at 405 nm. The % inhibition of the synthesized compounds and orlistat was calculated by using the following equation:

$$ {\text{Inhibitory}}\,{\text{lipase}}\,{\text{percentage}}\,\left( \% \right) = \left[ {\left( {A_{b} - \, A_{s} } \right)/A_{b} } \right]*100 $$

where *A*_*b*_ is blank absorbance, and *A*_*S*_ is sample absorbance. The IC_50_ values for tested synthetic compounds and orlistat were determined from the constructed curves.

### Molecular docking

The docking studies on the α-amylase enzyme were based on the human pancreatic amylase (the X-ray crystallographic structure (PDB code 4W93) that was obtained from the RCSB repository. This 3D protein structure was chosen based on its good resolution of crystallography (X-ray diffraction 1.352 *A*_*o*_). Additionally, this work is considered a continuation of our previous work on the α-amylase enzyme that utilized the ID: 4W93 α-amylase crystal structure^22^.

Preparing this crude obtained crystal structure for docking, using the PyMOL program (v1.8), started with removing the native ligand, non-protein atoms, and the present crystallographic waters. This step was followed by protonating imidazole and amide side chains via an H++ server. Then the polar hydrogen atoms were added and the structure was brought up to a physiological pH. The open-source program rDOCK (rdock.sourceforge.net), a development of RiboDock, was applied to perform the docking studies^23^. Considering the cavity occupied by the native ligand (a flavonol glycoside called montbretin A) of the utilized protein crystal structure (code 4W93), the binding site for docking studies was defined within 6A^o^ around it. The default docking procedure was followed that includes 3 stages of Genetic Algorithm search (GA-3, GA-2, and GA-1). This was followed by low-temperature Monte Carlo (MC) and Simplex minimization (MIN) stages^22^.

In the current study, the most potent compound against α-amylase was the **2a** compound**.** Applying the empirical score function of Rdock, the **2a** molecule was chosen to be docked in the binding pocket of the prepared crystal structure, keeping twenty docking solutions for each inhibitor to be sorted by their binding scores and later visually investigated for the connections between the inhibitors and the pocket’s residues.

### ADME-T calculations

A set of ADME-T related properties were calculated by using the QiKProp module (schrödinger 10.9, LLC, NY) running in normal mode. QikProp generates relevant descriptors and uses them to perform ADMET predictions^24,25^.

### Statistical analysis

The antidiabetic, antioxidant, and antilipase experimental works were conducted in triplicates and the results were expressed as means ± SD standard deviation while the result was considered significant when the *p *value was < 0.05.

## Results and discussion

### Antidiabetic activity

Hyperglycemia can be controlled by several therapeutic protocols, one of which is the inhibition of α-amylase and α-glucosidase metabolic enzymes^6,26^. In fact, α-amylase is a responsible enzyme for the hydrolysis of starch into simple monosaccharides^27–29^. While α-glucosidase enzyme can be found on the border of the small intestine and act upon hydrolysis of α-(1,4) bonds between monosaccharide units^30–32^.

**Figure 3:**
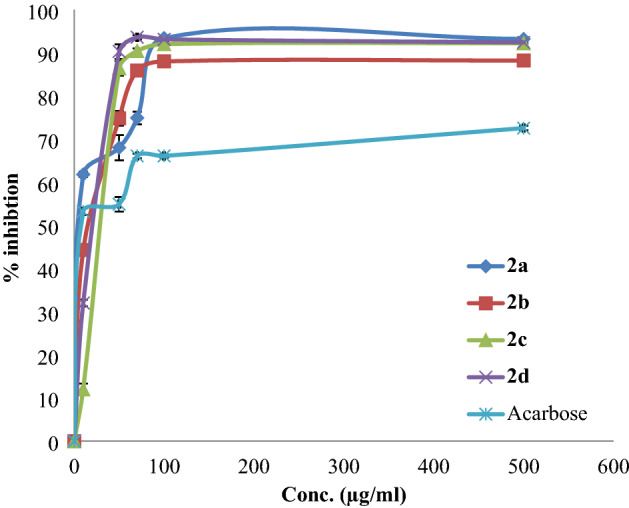
% Inhibition of α-amylase by the tested compounds.

**Figure 4:**
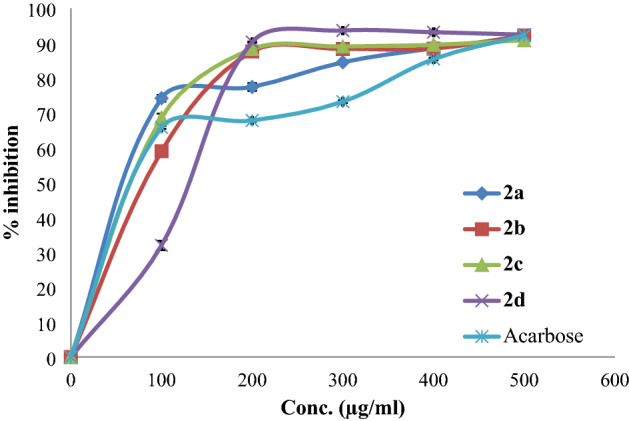
% Inhibition of α-glycosidase by the tested compounds.

The antidiabetic potential of the synthesized compounds was estimated by assessing their α-amylase and α-glycosidase inhibitory effects. Acarbose, which is a commercial antidiabetic drug, was used as a positive control. An evaluation of the plot of % α-amylase and α-glycosidase inhibition activities of each compound is shown in Figs. 3 and 4, respectively. The IC50 values were calculated (Table 1). All synthesized compounds show potential activity against the α-amylase enzyme.

**Table 1:** IC_50_ values of the synthesized compounds and acarbose regarding α-amylase and α-glycosidase inhibitory activities.

Compounds	α-Amylase enzyme IC_50_ (µg/ml), ± SD	α-Glycosidase enzyme IC_50_ (µg/ml), ± SD
**2a**	3.57 ± 1.08	10.09 ± 0.70
**2b**	12.44 ± 0.82	89.95 ± 0.49
**2c**	19.55 ± 0.96	78.1 ± 0.76
**2d**	13.41 ± 0.76	114.17 ± 1.04
**Acarbose**	6.47 ± 1.01	44.79 ± 0.33

Compound **2a** showed potent α-amylase inhibitory activity with an IC_50_ value of 3.57 ± 1.08 µg/ml in comparison with positive control acarbose (IC_50_ = 6.47 ± 1.01 µg/ml), as for compound **2a,** it demonstrated potent α-glycosidase inhibitory activity with an IC_50_ dose of 10.09 ± 0.70 µg/ml in comparison with acarbose (IC_50_ = 44.79 µg/ml). These results are reasonable and justified since the synthesized compounds are modified acid esters and have the same functional groups as phenolic compounds, such as trans-*p*-coumaric acid.

### Antioxidant activity

Antioxidants are valuable organic substances that can neutralize the harmful free radicals in living cells. There are two types of these radicals; natural pathways create the first type in our bodies due to the aging of cells and other factors; the second type is entered into our bodies from external sources like smoking, sun exposure, and other dangerous pollution sources. However, the body needs exogenous sources of synthetic antioxidants or dietary antioxidants from fruits and vegetables to scavenge these harmful radicals^6,33,34^.

### DPPH inhibitory activity

**Figure 5:**
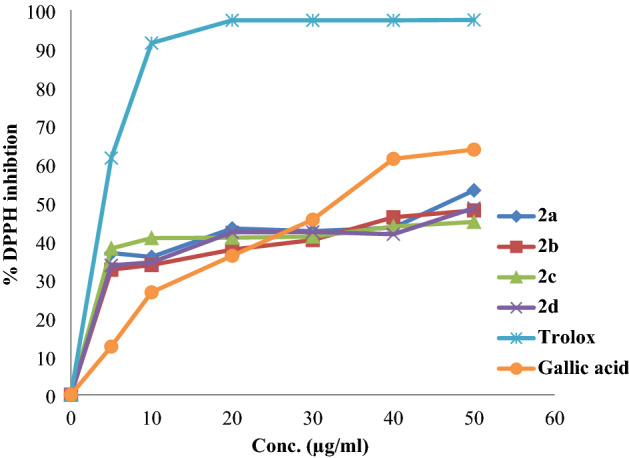
DPPH percent of inhibition by the tested compounds.

1,1-Diphenyl-2-picryl-hydroxyl (DPPH) is one of the most widely used methods for measuring the antioxidant activity of different compounds such as phenols and phenolic acid esters. The decrease in absorbance at 517 nm induced by antioxidants determines the reduction ability of the DPPH. When antioxidants donate hydrogen atoms to the radicals, they lose their purple color. This, in turn, leads to decreased absorption. The decrease in absorption is taken as a measure of the extent of radical scavenging. All the compounds showed free radical scavenging activity near gallic acid at a concentration of 30 µg/ml (Fig. 5). Compound **2a** showed potential antioxidant activity with an IC_50_ value of 47.01 ± 1.07 µg/ml compared with the positive controls, gallic acid and trolox, which have IC_50_ doses of 31.72 ± 1.22 and 4.28 ± 0.47 µg/ml, respectively (Table 2).

**Table 2:** Antioxidant activity IC_50_ values (µg/ml) of the synthesized compounds, trolox and gallic acid regarding the DPPH test.

Compounds	**2a**	**2b**	**2c**	**2d**	Trolox	Gallic acid
IC_50_ (µg/ml)	47.01 ± 1.07	50<	50<	50<	4.28 ± 0.47	31.72 ± 1.22

On the other hand, there is not much difference between percent inhibition values for the compounds. This means that the position of functional groups as ortho*-*, para- or meta*-* has a slight effect on the antioxidant activity. Our results are in agreement with literature done on polyphenolic compounds, which showed that the structure did not affect the antioxidant activity^35^.

### β-Carotene linoleic acid activity

**Figure 6:**
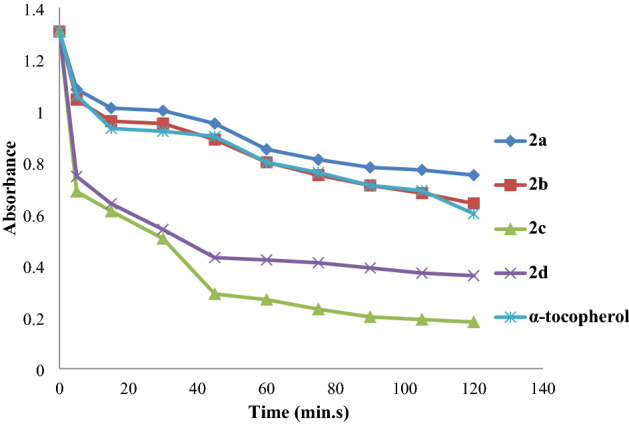
Antioxidant activities of synthesis compounds in *β*-carotene–linoleic acid test.

The same compounds were tested for their antioxidant activity using the emulsion system of β-carotene linoleic acid, depending on the fact that β-carotene loses its color in the absence of antioxidants. The synthetic compounds **2a** and **2b** showed higher antioxidant efficiency in concentration 0.2 mg/ml compared with water (control) and α-tocopherol and the other synthetic compounds antioxidant, which gave the lowest antioxidant efficiency. Figure 6 showed the antioxidant activities of the synthetic acid esters and positive reference standard with the coupled oxidation of β-carotene in a concentration of 0.2 mg/ml.

The antioxidant activity of all the compounds gradually decreases with the increase of time. Water showed the highest β-carotene bleaching activity, followed by α-tocopherol after 1 h and (**2a**) compound, which has the best antioxidant activity with the absorbance of 1.04 after 75 min compared with the other synthetic tested compounds. The second one is a **2b** compound which showed absorbance 0.84 after 75 min.

### Porcine pancreatic lipase enzyme activity

**Figure 7:**
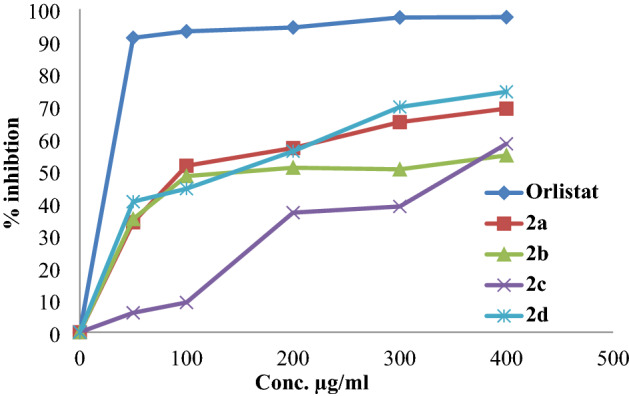
Lipase inhibition % by the synthesized compounds and orlistat.

The hydrolysis of *p*-nitrophenyl butyrate to *p*-nitrophenyl was used to measure the influence of the synthetic acid esters on the porcine pancreatic lipase enzyme. The assay measures the inhibition percentages of the synthesized compounds and the obtained results compared with orlistat, a pharmaceutical anti-obesity drug, and used in the current study as a positive control. The results of inhibitory percentages for the compounds and orlistat are shown in Fig. 7.

The anti-lipase activity IC_50_ values of the synthesized compounds were with the range of 107.25–341.06 µg/ml in comparison with orlistat (IC_50_ = 25.01 ± 0.78 µg/ml). The best inhibition activity was observed in compounds **2a** and **2d** with IC_50_ values of 107.95 ± 1.88 and 119.05 ± 2.04 µg/ml, respectively. Compound **2c** has the lowest inhibitory activity with an IC_50_ value of 341.06 ± 2.40 µg/ml.

### Docking study

**Figure 8:**
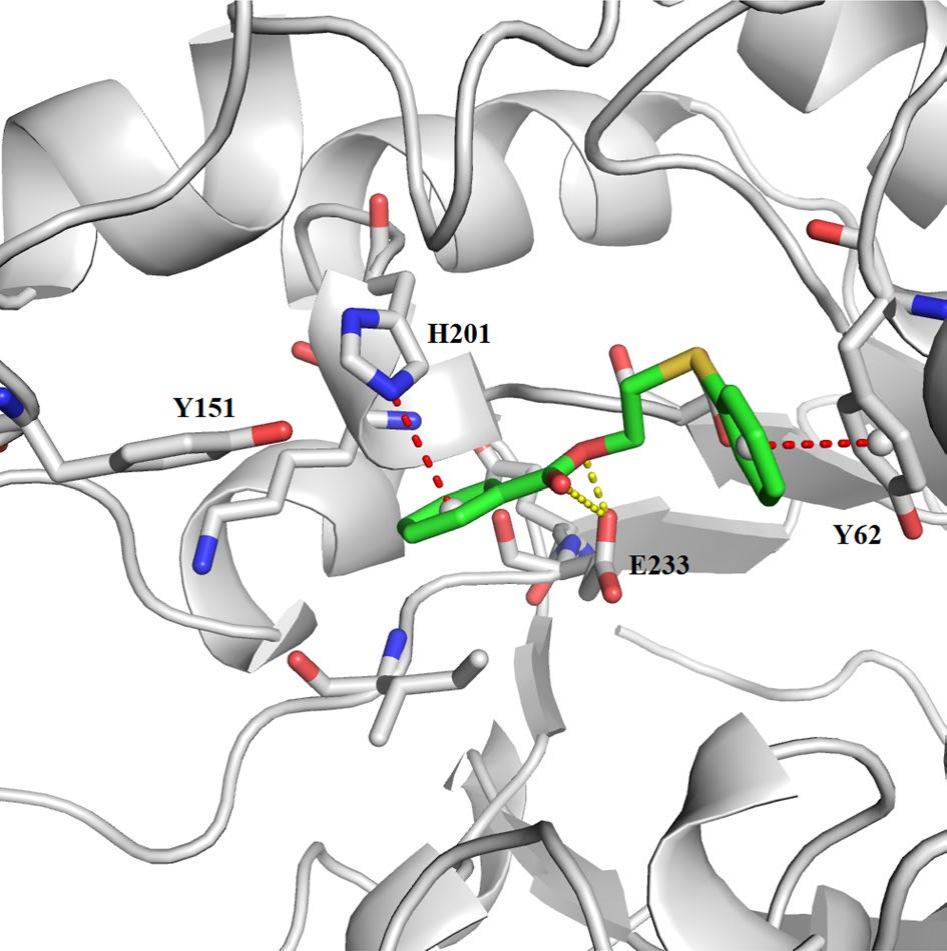
Docking solution of the compound **2a** inside the binding pocket of α-amylase (pdb code: 4w93) forming hydrogen bonds with the residue E233 and charge–π interactions with H201.

The **2a** compound, which exhibited a super anti-diabetic and anti-lipase activity besides super antioxidant activity (considering the β-Carotene method) over the other designed compounds, was docked to the binding pocket of the α-amylase protein structure ID: 4W93). This docking revealed the formation of hydrogen bonds with the residue E233 and charge–π interactions with H201; stabilized by interactions with other surrounding residues like π–π stacking with Y62. Our docking studies suggest the potent inhibition of α-amylase is derived from strong interactions with the catalytic residue E233 and the neighbor residue H201, besides other stabilizing interactions with surrounding residues (Fig. 8) as well as the molecular Docking scores and α-amylase inhibtory activities are presented in Table 3.

**Table 3:** The molecular Docking score of the 2-(phenylthio)-ethyl benzoate derivatives inside α-amylase crystal structure (PDB code: 4w93) within 6 Å around the binding pocket.

Compounds	**2a**	**2b**	**2c**	**2d**
α-Amylase enzyme IC_50_ (µg/ml)	3.57 ± 1.08	12.44 ± 0.82	19.55 ± 0.96	13.41 ± 0.76
Score (rDOCK)	− 11.55	− 9.95	− 8.89	− 10.41
Score.inter	− 12.08	− 11.35	− 11.29	− 11.54
Score.intra	1.15	1.83	1.67	1.98
Score.rest	0	0	0	0
Score.site	− 0.62	− 0.43	− 0.24	− 0.85

The rDock master scoring function (Score) is a sum of intermolecular (Score.inter), ligand intramolecular (Score.intra), site intramolecular (Score.site), and external restraint terms (Score.restraint). [rDOCK manual: see http://rdock.sourceforge.net/documentation/].

### ADME-T calculations

All the calculated ADME-T parameters of our new compounds and the ideal recommended values are summarized in Table 4. It was clear that all synthesized compounds are located in the recommended and ideal range and do not exhibit any violations outside the recommended ADMET values.

**Table 4:** The ADME properties of molecules **2a, 2b, 2c**, and **2d** using QiKProp module (schrödinger 10.9, LLC, NY) running in normal mode.

		Compounds				Recommended values
		**2a**	**2b**	**2c**	**2d**	
ADMET parameters	Mol_MW	258.334	278.334	278.334	278.334	130–725
	Dipole	3.823	2.202	3.180	1.282	1.0–12.5
	QPlogPoct++	11.033	10.857	13.055	12.928	8.0–35
	QPlogPo/w	4.079	4.057	3.443	3.443	− 2.0–6.5
	QPlogS	− 4.215	− 4.468	− 4.216	− 4.216	− 6.0–0.5
	QPlogBB	− 0.090	− 0.528	− 0.704	− 0.704	− 3–1.2
	QPlogKhsa	0.298	0.388	0.216	0.216	− 1.5–1.5
	Percent human oral absorption	100	100	100	100	> 80% is high < 25% is low
	PSA	33.4	51.9	56.0	56.0	7–200
	Rule of five	0	0	0	0	< 4
	Rule of three	0	0	0	0	< 3

Mol Mw: molecular weight of the molecule. Dipole: computed dipole moment of the molecule. QPlogPoct++: predicted octanol/gas partition coefficient. QPlogPo/w: predicted octanol/water partition coefficient. QPlogS: predicted aqueous solubility, S in mol dm^−3^. QPlogBB: predicted brain/blood partition coefficient. QPlogKhsa: prediction of binding to human serum albumin. Percent Human Oral Absorption: predicted human oral absorption on a 0–100% scale. PSA: van der Waals surface area of polar nitrogen and oxygen atoms and carbonyl carbon atoms. Rule of five: number of violations of Lipinski rule of 5. Rule of three: number of violations of Jorgensen’s rule of 5.

De facto, diabetes mellitus is a growing health problem worldwide without therapeutic agents that can control glucose levels in the bloodstream, leading to many macroangiopathic complications, including neuropathy, glaucoma, carpal tunnel syndrome, diabetic ulcers, atherosclerosis phenomena, and many others. In addition, diabetes also can cause microangiopathies injuries, including neuropathies of the peripheral nervous system, retinopathies, and nephropathies. In addition, it can increase the risk of various infectious diseases and cancer^1,36–38^. Therefore, the search for practical and multi-functional drugs that can treat diabetes, obesity, and oxidative stress at the same time must be one of the main goals of modern scientists worldwide.

## Conclusion

The results of the current investigation revealed that synthetic compound **2a** exhibited potential anti-diabetic and anti-obesity properties. The **2a** compound has strong potential in the inhabitation of metabolic key enzymes, including α-amylase, α-glycosidase, and anti-lipase, compared with the positive controls. Moreover, the **2a** molecule demonstrated a potential antioxidant effect against DPPH and β-carotene linoleic acid-free radicals. The discovery of this simple synthetic compound may lead to the emergence of a new generation of antidiabetic, antioxidant, and anti-obesity compounds that can be used to manufacture multi-target medicines based on benzoic acid derivatives. Thus, studying the properties of these bioactive molecules will contribute to combating these dangerous disorders and limiting their repercussions in the world. Moreover, the current study results may encourage other researchers to conduct more studies on these benzoic acid derivatives.

## Data Availability

The datasets used and/or analyzed during the current study available from the corresponding author on reasonable request.

## Abbreviations

DPPH: 2,2-Diphenyl-1-picrylhydrazyl
IC_50_: Half maximal inhibitory concentration
WHO: World Health Organization
DNSA: 3,5-Dinitrosalicylic acid
DMSO: Dimethyl sulfoxide
PNPG: *p*-Nitrophenyl glucopyranoside
Trolox: ((S)-(–)-6 hydroxy-2,5,7,8-tetramethychroman-2-carboxylic acid)
PNPB: *p*-Nitrophenyl butyrate

## References

[1] Cervino G (2019). Diabetes: Oral health related quality of life and oral alterations. Biomed. Res. Int..

[2] Jeong S (2019). Chitosan oligosaccharide (GO2KA1) improves postprandial glycemic response in subjects with impaired glucose tolerance and impaired fasting glucose and in healthy subjects: A crossover, randomized controlled trial. Nutr. Diabetes.

[3] Mosleh R, Hawash M, Jarrar Y (2020). The relationships among the organizational factors of a tertiary healthcare center for type 2 diabetic patients in Palestine. Endocr. Metab. Immune Disord. Drug Targets.

[4] Diplock A (1998). Functional food science and defence against reactive oxidative species. Br. J. Nutr..

[5] Davies K, Pryor W (2005). The evolution offree radical biology & medicine: A 20-year history. Free Radical. Bio. Med..

[6] Kanwal (2021). Indole-3-acetamides: As potential antihyperglycemic and antioxidant agents; synthesis, in vitro α-amylase inhibitory activity, structure–activity relationship, and in silico studies. ACS Omega.

[7] Taha M (2020). Synthesis, antiglycation and antioxidant potentials of benzimidazole derivatives. J. King Saud Univ. Sci..

[8] Hawash M (2019). Highlights on specific biological targets; cyclin-dependent kinases, epidermal growth factor receptors, ras protein, and cancer stem cells in anticancer drug development. Drug Res..

[9] Sharma H, Chandola H (2011). Ayurvedic concept of obesity, metabolic syndrome, and diabetes mellitus. J. Altern. Complement Med..

[10] Yazdi FT, Clee SM, Meyre D (2015). Obesity genetics in mouse and human: Back and forth, and back again. PeerJ.

[11] Hawash MM, Baytas SN (2017). Antiproliferative activities of some biologically important scaffold. Fabad J. Pharm. Sci..

[12] Jaradat N (2020). In vitro antitumor, antibacterial, and antifungal activities of phenylthio-ethyl benzoate derivatives. Arab. J. Sci. Eng..

[13] Wickramaratne MN, Punchihewa J, Wickramaratne D (2016). In-vitro alpha amylase inhibitory activity of the leaf extracts of *Adenanthera pavonina*. BMC Complement Med. Ther..

[14] Hawash M (2019). Evaluation of the hypoglycemic effect of seven wild folkloric edible plants from Palestine. J. Complement. Integr. Med..

[15] Ullah H (2020). Aryl-oxadiazole Schiff bases: Synthesis, α-glucosidase in vitro inhibitory activity and their in silico studies. Arab. J. Chem..

[16] Burits M, Bucar F (2000). Antioxidant activity of Nigella sativa essential oil. Phytother. Res..

[17] Gazzani G (1998). Anti-and prooxidant activity of water soluble components of some common diet vegetables and the effect of thermal treatment. J. Agr. Food Chem..

[18] Jayaprakasha GK, Singh R, Sakariah K (2001). Antioxidant activity of grape seed (*Vitis vinifera*) extracts on peroxidation models in vitro. Food Chem..

[19] Bustanji Y (2011). Pancreatic lipase inhibition activity of trilactone terpenes of *Ginkgo biloba*. J. Enzyme Inhib. Med. Chem..

[20] Jaradat NA, Zaid AN, Hussein F (2016). Investigation of the antiobesity and antioxidant properties of wild *Plumbago europaea* and *Plumbago auriculata* from North Palestine. Chem. Biol. Technol. Agric..

[21] Keskes H (2014). In vitro anti-diabetic, anti-obesity and antioxidant proprieties of *Juniperus phoenicea* L. leaves from Tunisia. Asian Pac. J. Trop. Biomed..

[22] Hawash M (2021). Molecular docking, chemo-informatic properties, alpha-amylase, and lipase inhibition studies of benzodioxol derivatives. BMC Chem..

[23] Morley SD, Afshar M (2004). Validation of an empirical RNA-ligand scoring function for fast flexible docking using RiboDock^®^. J. Comput. Aided. Mol. Des..

[24] Wu Y (2021). Polyphenols as alternative treatments of COVID-19. Comput. Struct. Biotechnol. J..

[25] Taslimi P (2021). The biological activities, molecular docking studies, and anticancer effects of 1-arylsuphonylpyrazole derivatives. J. Biomol. Struct. Dyn..

[26] Saleem F (2021). Synthesis of azachalcones, their α-amylase, α-glucosidase inhibitory activities, kinetics, and molecular docking studies. Bioorg. Chem..

[27] Hill R (1970). The chemistry of life: Eight lectures on the history of biochemistry.

[28] Stenesh J (1998). Biochemistry 2.

[29] Hussain S (2021). Synthesis of benzimidazole derivatives as potent inhibitors for α-amylase and their molecular docking study in management of type-II diabetes. J. Mol. Struct..

[30] Rauf, A. & Jehan, N. Natural products as a potential enzyme inhibitors from medicinal plants, in *Enzyme Inhibitors and Activators *165–177 (InTech Rijeka, 2017).

[31] Sorensen SH (1982). Amphiphilic pig intestinal microvillus maltase/glucoamylase: Structure and specificity. Eur. J. Biochem..

[32] Taha M (2021). Evaluation and docking of indole sulfonamide as a potent inhibitor of α-glucosidase enzyme in streptozotocin–induced diabetic albino wistar rats. Bioorg. Chem..

[33] Hussein, A. I. A., *Modification of biologically active compounds from selected medicinal plants in Palestine*. 2009.

[34] Halliwell, B. & J. M. Gutteridge, *Free radicals in biology and medicine.* 1985. 10.1016/0748-5514(85)90028-5 3939136

[35] Vitalone A (2003). Extracts of various species of Epilobium inhibit proliferation of human prostate cells. J. Pharm. Pharmacol..

[36] Babatunde, O., et al., *Dihydroquinazolin-4 (1H)-one derivatives as novel and potential leads for diabetic management *1–20 (Mol Divers, 2021). 10.1007/s11030-021-10196-5 33650031

[37] Takao T (2020). Combined effect of diabetic retinopathy and diabetic kidney disease on all-cause, cancer, vascular and non-cancer non-vascular mortality in patients with type 2 diabetes: A real-world longitudinal study. J. Diabetes Investig..

[38] Zoppini G (2018). Mortality from infectious diseases in diabetes. Nutr. Metab. Cardiovasc. Dis..

